# The Presence of Circulating Nucleated Red Blood Cells Is Associated With Disease Severity in Patients of Hemorrhagic Fever With Renal Syndrome

**DOI:** 10.3389/fmed.2021.665410

**Published:** 2021-05-25

**Authors:** Jingang Zhang, Kang Tang, Yun Zhang, Ying Ma, Chunmei Zhang, Haifeng Hu, Xiaozhou Jia, Ran Zhuang, Boquan Jin, Meng Wang, Xiyue Zhang, Dalu Liu, Yusi Zhang

**Affiliations:** ^1^Department of Immunology, Basic Medicine School, Air Force Medical University, Xi'an, China; ^2^Brigade of Cadet, Air Force Medical University, Xi'an, China; ^3^Center for Infectious Diseases, Second Affiliated Hospital of Air Force Medical University, Xi'an, China; ^4^Department of Infectious Disease, Xi'an Eighth Hospital, Xi'an, China; ^5^Department of Radiation Medicine and Protection, Ministry of Education Key Lab of Hazard Assessment and Control in Special Operational Environment, School of Public Health, Air Force Medical University, Xi'an, China

**Keywords:** HTNV, HFRS, NRBC, vitamin B12 (B12), folic acid (B9)

## Abstract

Hemorrhagic fever with renal syndrome (HFRS) is a regional infectious disease of epidemic potential caused by the Hantaan virus (HTNV). Red blood cells (RBCs) are the major components of peripheral blood. However, pathological changes in RBCs and the underlying mechanisms during HTNV infection remain largely unclear. Therefore, this study sought to explore changes in RBCs in the peripheral blood of HFRS patients. We isolated PBMCs from HFRS patients and performed single-cell RNA sequencing. The results showed that clusters of RBCs in the peripheral blood of HFRS could be classified as nucleated red blood cells (NRBC) based on their cellular components, gene expression profiles and cell surface markers. In addition, it was shown that the higher the count of NRBC in peripheral blood, the more severe the disease status was. Moreover, hematological indices related to RBCs were analyzed and the results showed that impairment in the folate pathway might be the possible reason behind the presence of NRBCs. This study, for the first time showed that the presence of NRBCs in the peripheral blood of HFRS patients was associated with disease severity. This was also the first study to show that infection with the HTNV virus hindered the maturation of RBCs. Therefore, this work provides further insights on the role of and pathological changes in RBCs during HTNV infection.

## Introduction

Hemorrhagic fever with renal syndrome (HFRS) is caused by infection with the Hantaan virus (HTNV) and has resulted to epidemics throughout Eurasia. Notably, China has the highest number of HFRS patients worldwide (1). Moreover, the phenotypic and functional characteristics of peripheral blood mononuclear cells (PBMCs) including T cells, B cells, dendritic cells and natural killer cells have been well-studied during HTNV infection (2–6). However, responses in red blood cells (RBCs), a major component of blood to HTNV infection are yet to be reported.

RBCs are developed within the bone marrow through the process of erythropoiesis which involves a series of maturation steps. During this process, multipotent hematopoietic stem cells differentiate into erythroid progenitor cells. Thereafter, nucleated precursors differentiate from proerythroblasts into basophilic, polychromatic and orthochromatic erythroblasts (7, 8). These stages result to the accumulation of hemoglobin (HGB) (9). Additionally, when reticulocytes enter peripheral circulation, they undergo enucleation, loss of organelles and subsequent maturation into RBCs (10). Therefore, given the maturation process of RBCs, the nucleated red blood cells (NRBCs) should remain in the bone marrow of healthy adults except for certain physiological or pathological conditions where they become visible.

Studies on intensive care unit (ICU) patients reported that the appearance of NRBC in circulation was a predictor of increased mortality and poor prognosis (11–13). Moreover, the role of NRBCs in some infectious diseases such as HIV was reported. The immature RBCs (CD71^+^erythroid cells) were found to contribute to the persistence and transmission of HIV (14). However, none of these studies explored the gene expression profile of the NRBCs and their underlying physiological functions. Furthermore, little is known about the causes of the presence of NRBCs in peripheral blood under disease conditions.

In order to address these gaps, the present study for the first time performed single-cell RNA sequencing (scRNA-seq) to profile the PBMCs of HFRS patients. The results showed that NRBCs were present in circulation during infection with HTNV. Moreover, the study established that the presence of NRBCs was associated with disease severity by evaluating the cell counts, clusters distributions and gene expression of the cells. Furthermore, reasons behind the appearance of NRBCs were explored and the study established that disorders in the folate pathway might contribute to the appearance of the cells in the peripheral blood of HFRS patients. Therefore, this study not only depicts the cellular components and gene expression profiles of NRBCs in the circulating blood of HFRS patients but also explores the possible reasons behind these phenomena. Consequently, the results will help in understanding how the erythroid cell lineage copes with the challenge of HTNV infection.

## Materials and Methods

### Sample Collection

All the HFRS patients enrolled in this study were recruited from the Tangdu Hospital of the Fourth Military Medical University (Xi'an, China) and Xi'an Eighth Hospital from 2019 to 2021. Clinical diagnosis of HFRS was confirmed serologically through the detection of specific IgM and IgG antibodies against the HTNV-nucleocapsid protein (NP). The peripheral whole blood samples were collected using vacuum-based blood collectors with ethylenediaminetetraacetic acid (EDTA) from 82 HFRS patients (62 males and 20 females within the age range of 16–85 years) during hospitalization. Following centrifugation at 3,000 rpm for 15 min, the plasma layer was aliquoted and stored at −80°C until use. Additionally, PBMCs were isolated by standard Ficoll density gradient centrifugation and frozen in liquid nitrogen with RPMI/10% DMSO as the freezing solution and used within 2 weeks. The viral load in each sample was measured according to a method previously established in our lab (15). All procedures followed were in accordance with the ethical standards of the responsible committee on human experimentation [Xijing Hospital, First Affiliated Hospital of Fourth Military Medical University, Xi'an, China, (NO. KY20173177-1)] and with the Helsinki Declaration of 1975, as revised in 2008. Informed consent was obtained from all patients for being included in the study. One child patient was included with the written consent obtained from a parent and assent obtained from the child patient herself. All the data were analyzed anonymously.

### Single-Cell RNA Sequencing

All the sequencing was done by the Novel Bioinformatics Co., Ltd, Shanghai, P. R. China. Briefly, the single-cell RNA sequencing (scRNA-Seq) libraries were generated using the 10X Genomics Chromium Controller Instrument and Chromium Single Cell 5' library & gel bead kit (10X Genomics, Pleasanton, CA). The cells were then concentrated to 1,000 cells/μL and ~6,000 cells were loaded into each channel to generate single-cell gel bead-in-emulsions (GEMs). Following the RT step, GEMs were broken and the barcoded-cDNA purified and amplified. The amplified barcoded cDNA was then used to construct 5' gene expression libraries. Afterwards, the amplified barcoded cDNA was fragmented, A-tailed, ligated with adaptors and index PCR amplified. The final libraries were quantified using the Qubit High Sensitivity DNA assay (Thermo Fisher Scientific) and the size distribution of the libraries determined using a High Sensitivity DNA chip on the Bioanalyzer 2,200 (Agilent). All libraries were sequenced using the illumina sequencer (Illumina, San Diego, CA) on a 150 bp paired-end run.

### Single-Cell RNA Statistical Analysis

scRNA-seq data analysis was performed by the Novel Bioinformatics Co on the NovelBrain Cloud Analysis Platform. The adaptor sequence was filtered and low-quality reads removed to obtain clean data. Afterwards, the feature-barcode matrices were obtained by aligning reads to the human genome (GRCh38 Ensemble: version 91) using cellranger v3.1.0. Cells that contained over 200 expressed genes and had a mitochondria UMI rate below 20% passed the cell quality filtering and mitochondrial genes were removed from the expression table.

Moreover, the Seurat package (version: 2.3.4) was used for cell normalization. Regression was based on the expression table according to the UMI counts of each sample and percent of mitochondrial rate to obtain the scaled data. Principal component analysis (PCA) was constructed based on the scaled data with the top 2,000 highly variable genes. In addition, the top 10 principals were used for constructing the t-distributed stochastic neighbor embedding (tSNE) graph. Using the graph-based cluster method, the unsupervised cell cluster result was obtained based on the PCA top 10 principals. Moreover, the marker genes were calculated using the Find All Markers function and the wilcox rank sum test algorithm utilizing the following criteria: 1. lnFC>0.25; 2. *P*-value<0.05; 3. min.pct>0.1. Finally, in order to obtain details on the cell types, clusters of the same cell type were selected for re-tSNE analysis, graph-based clustering and marker analysis.

### Pseudotime Analysis

Single-cell trajectories analysis was performed through Monocle2 (http://cole-trapnell-lab.github.io/monocle-release) using the DDR-Tree and default parameters. Before Monocle analysis, the marker genes from the Seurat clustering results were selected and raw expression counts of the cell passed filtering. Based on pseudotime analysis, branch expression analysis modeling (BEAM Analysis) was applied for branch fate determined gene analysis.

### Co-regulated Gene Analysis

The find_gene_modules function of Monocle3 was used with the default parameters to discover the gene co-regulation network.

### *In vitro* HTNV Infection

Peripheral whole blood samples were collected using vacuum-based blood collectors with EDTA from 4 health donors. Following centrifugation at 3,000 rpm for 15 min, the plasma layer was aliquoted and stored at −80°C until use. The whole blood cells were resuspended in RPMI 1,640 with 10% FBS.

HTNV strain 76–118 was stored and frozen at −80°C in the Department of Microbiology, Fourth Military Medical University. Mock HTNV control was prepared by incubating HTNV at 60 °C for 30 min. For the infection, virus was allowed to adsorb to the cells at multiplicity of infection (MOI) of approximately 1 for 2 h at 37 °C. The cells were then washed and afterwards incubated in RPMI 1,640 with 10% FBS. After 72 h post infection, the cells were collected.

### Flow Cytometry Analysis

For PBMCs surface staining, 4×10^6^ freshly isolated PBMCs from 40 HFRS patients in different disease severity and 7 health donors were used. For whole blood cell staining, 100 μl whole blood cells from 4 health donors were used. The cells were stained with anti-CD71 antibody (eBioscience, clone: OKT9) in staining buffer (PBS containing 3% FCS and 0.01% NaN3) for 30 min on ice at first place. After washing the cells with staining buffer once, the Alexa fluor 488-conjugated goat anti-mouse antibody (Invitrogen) was used as a secondary antibody. After incubating for 30 min on ice, the cells were washed again with staining buffer and stained with the PE-CD235a (BioLegend, clone: H1264), APC-Cy7-CD45 (BioLegend, clone: 2D1) and Alexa fluor 647-αvβ3 (BioLegend, clone: 23C6) were added and incubated for another 30 min on ice. The cells were then washed once with cold staining buffer before re-suspending in staining buffer. The intracellular staining of HTNV-NP was performed as follows. 100 μl whole blood cells were performed surface staining with APC-Cy7-CD45, APN-CD71 (BioLegend, Clone: CY1G4) and PE-CD235a firstly. Intracellular staining with FITC-NP (clone: 1A8, produced by Department of Microbiology, Fourth Military Medical University) (16) was performed after fixating and permeabilizing the cells using an intracellular staining kit (eBioscience Fixation/Permeabilization kit). All procedures were performed according to the manufacturer's instructions. Flow cytometry was conducted on an ACEA *Novo* Express system (Agilent Bio) and data analyzed using the FlowJo software (TreeStar). Results were expressed as a percentage of positive cells.

### Enzyme-Linked Immunosorbent Assay (ELISA)

Competitive ELISA assays for vitamin B12 (Catalog No. EHC9140) and folate (Catalog No. EHC9139) were performed using ELISA kits from Neobioscience according to the manufacturer's protocol. Absorbance at 450 nm was then obtained using the SpectraMax Reader (Molecular devices).

### RNA Extraction and Real-Time PCR

The RBCs were enriched by sorting CD235a^+^ cells. The total RNA of RBCs was extracted using TRIzol (Invitrogen) according to the manufacturer's protocol and 1 μg was used for cDNA synthesis (Takara, Japan). Quantitative analysis of mRNA expression was done by quantitative Real-time PCR using the SYBR Green detection method. The specific primers for *NP* and *β-actin* were as follows. *NP* (F: TACAGAGGGAAATCAATGCC; R: TGTTCAACTCATCTGGATCCTT), *β-actin* (F: CATGTACGTTGCTATCCAGGC; R: CTCCTTAATGTCACGCACGAT) Reactions were analyzed using a BIO-RAD system (CFX96 Real-Time System). The delta ct method was used to calculate. Then, *NP* was normalized to the housekeeping gene *β-actin* and was presented as fold changes of untreated cells.

### Statistical Analysis

Prior to the analyses, all the data was tested for normality using the Shapiro-Wilks test and homogeneity of variance using the Levenes test. Thereafter, One-way ANOVA and student *t*-test were used for comparison between the groups. In addition, correlation coefficients were computed using Pearson correlation or Spearman correlation. All the statistical tests were two sided and *P*-values < 0.05 were considered significant.

## Results

### Red Blood Cells Shown in PBMCs of HFRS Patients

**Figure 1:**
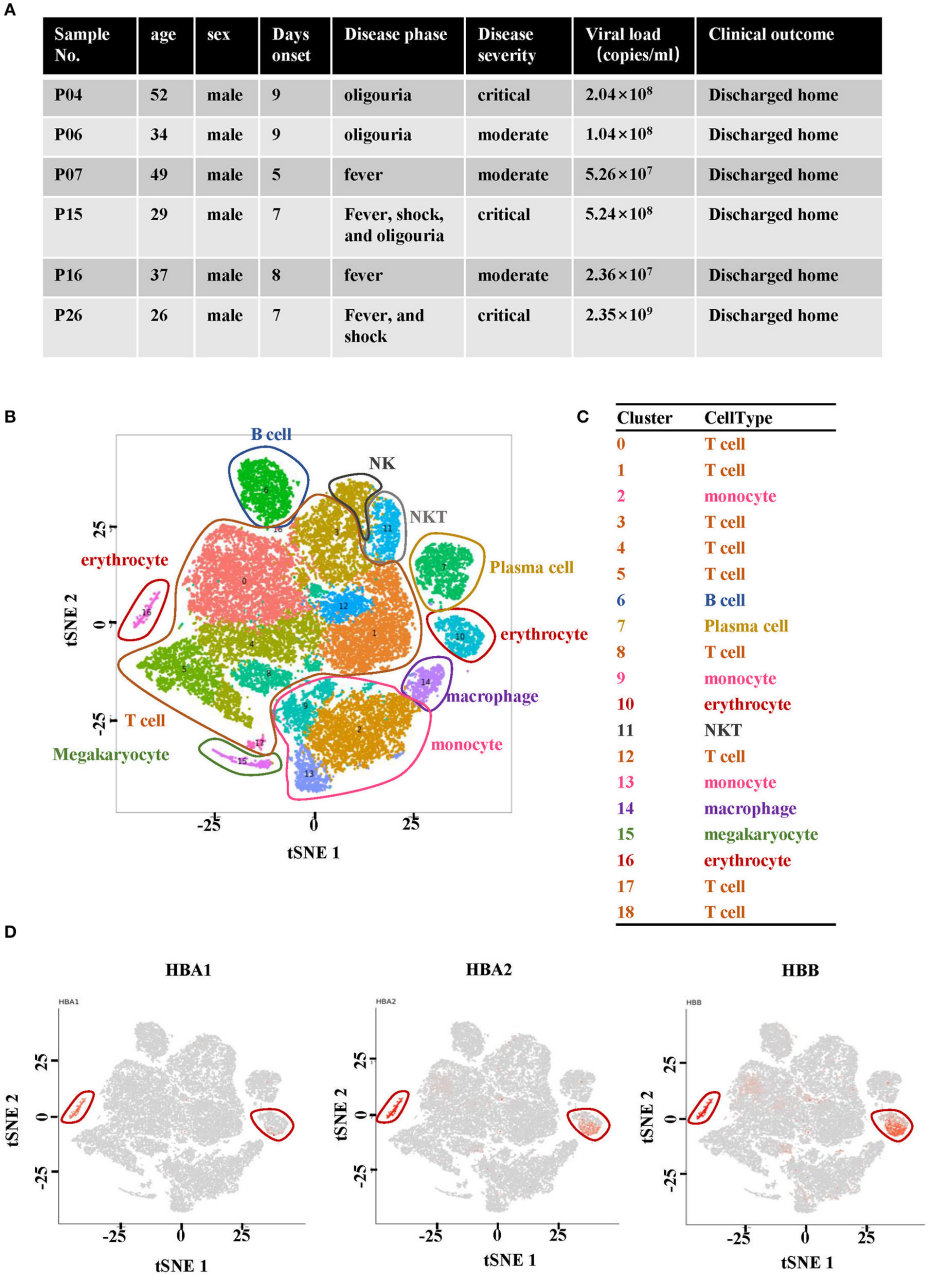
Single-cell RNA sequencing analysis of PBMCs from HFRS patients. **(A)** Characteristics of the samples and the disease course of patients with HFRS. **(B)** The tSNE presentation of the cell types and **(C)** associated clusters in PBMC from HFRS patients. **(D)** The expression of HBA1, HBA2 and HBB in cluster 10 and 16 defined the two clusters as erythrocytes.

In order to profile the peripheral immune response to HTNV, scRNA-seq was performed on isolated PBMCs from two healthy donors and six hospitalized patients. The patients had been diagnosed as HFRS positive using the anti-HTNV IgM. The demographics and clinical features of the patients are listed in Figure 1A. All the six patients profiled were male and aged between 26 and 52 years. On the other hand, the healthy donors consisted of individuals working in our lab. One was a 29-year-old male while the other was a 33-year-old female. The samples were collected within 10 days after the onset of disease to ensure that all the patients were in the acute phase of HFRS which included fever, shock, and diuretic stages. With regard to severity, patients P04, P15 and P26 were diagnosed as critical while the other three, P06, P07 and P16 were found to have moderate severity (Figure 1A).

The cells sequenced in each sample are summarized in Supplementary Table 1. The tSNE graph partitioned cells into 19 clusters (Figure 1B). In addition, the regular PBMCs were identified as T cells (Cluster 0, 1, 3–5, 8, 12, 17, and 18), B cells (cluster 6), monocytes (cluster 2, 9, and 13), plasma cells (cluster 7), NKT cells (cluster 11), macrophages (cluster 14) and megakaryocyte (cluster 15). Erythrocytes (red blood cells, RBCs, cluster 10 and 16) were also identified (Figures 1B,C). The characteristic genes of RBCs in cluster 10 were hemoglobin B (*HBB*) and hemoglobin A2 (*HBA2*) while those in cluster 16 were *HBA1*, hemoglobin A2 (*HBA2*) and *HBB* (Figure 1D). The other marker genes expressed in cluster 10 and cluster 16 are shown in Supplementary Figures 1, 2.

**Figure 2:**
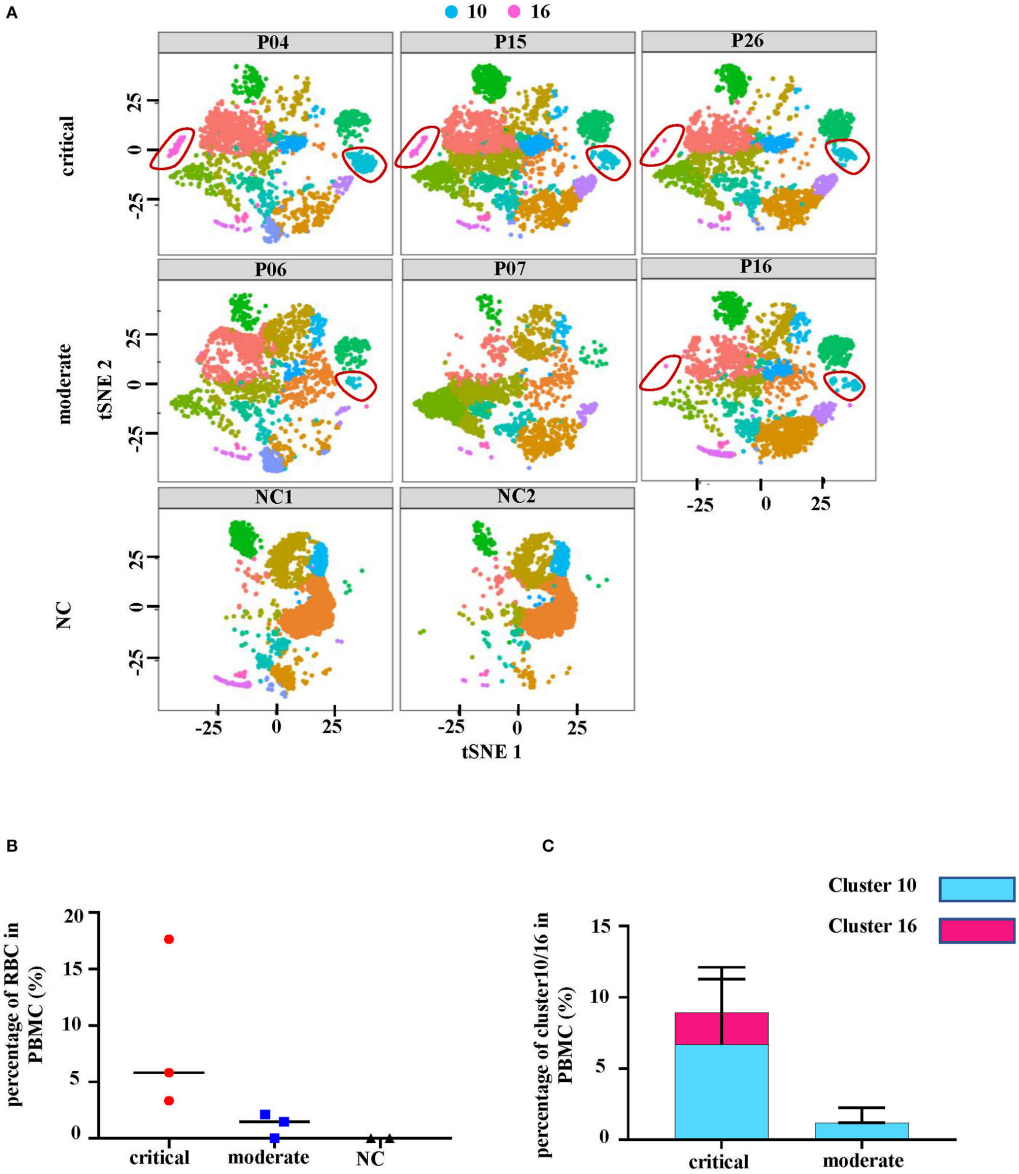
The number of erythrocytes was associated with disease severity. **(A)** The tSNE plots of each studied subject, including 3 critical cases (P04, P15 and P26), 3 moderately severe cases (P06, P07, and P16) and 2 controls (NC1 and NC2). **(B)** The percentages of erythrocytes (red blood cells, RBC) in all the cells as shown in different levels of disease severity. **(C)** The comparison of cluster 10 and cluster 16 proportions in different levels of disease severity.

The distribution of RBCs in each studied sample was then examined. It was observed that RBCs were absent in the two healthy donors and P07, one of the HFRS patients with moderate severity (Figure 2A). The other two samples from moderate HFRS patients, P06 and P16, contained RBCs majorly in cluster 10. Additionally, the percentage of absolute cell counts of RBCs in the total sequenced cells were calculated (Figure 2B). There is a trend that the critical HFRS patients had more RBCs in PBMCs (3.3–17.6%) than those with moderate HFRS (1.5~2.1%) and healthy donors (0%) (Figure 2B). Moreover, the percentages of total sequenced cells in cluster 10 and 16 in HFRS patients with different levels of severity are shown for comparison. Clusters10 and 16 both had critical HFRS patients while only cluster 10 had HFRS patients with moderate severity (Figure 2C). Therefore, these results indicated that the RBCs mainly appeared in the critical HFRS patients. However, although some of the moderate HFRS patients had RBCs, most of the cells were classified under cluster 10.

### The RBCs Were Characterized as Nucleated Cells

**Figure 3:**
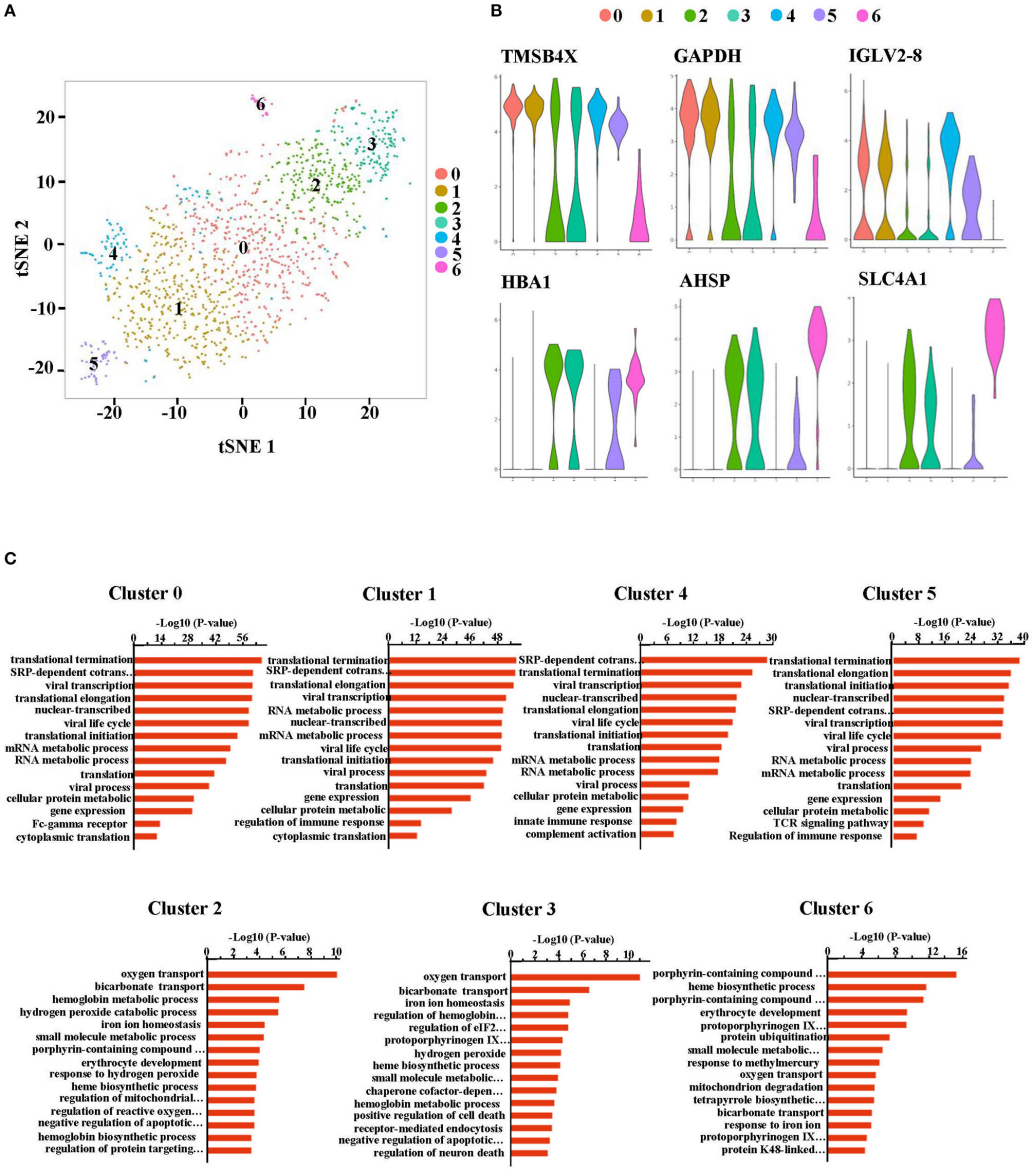
The RBCs in the peripheral blood from HFRS patients were defined as nucleated red blood cells (NRBCs). **(A)** Erythrocytes (cluster 10 and cluster 16) were taken out and reanalyzed. tSNE projection revealed 7 new clusters (cluster 0–6). **(B)** Representative violin plot of single-cell expression levels of the characteristic genes in each cluster. The progenitor-like genes were enriched in cluster 0, 1, 4, and 5 (upper). The erythroblast-related genes were enriched in the cluster 2, 3, and 6 (lower). **(C)** Bar charts depicting gene ontology (BP) enrichment analysis (biological process: BP). Each row represents a pathway.

A more specific analysis on RBCs was then conducted and they were further separated into seven new clusters (Figure 3A). The heatmap in Supplementary Figure 3 shows the signature genes in each cluster. Clusters that had similar gene expression profiles were grouped close together. Generally, clusters 0, 1, 4 and 5 were grouped as a module while clusters 2, 3, and 6 were classified as another module (Supplementary Figure 4). Clusters 0, 1, 4, and 5 expressed genes related to the hematopoietic system (such as *TMSB4X*), nuclear functions (such as *GAPDH*) and BCR or TCR rearrangement (such as *IGLV2-8*) (Figure 3B). On the other hand, clusters 2, 3, and 6 had genes related to the functioning of mature erythrocytes (such as *HBA1*) and intermediate stages during normal erythroid cell development (such as *AHSP* and *SLC4A1*) (17) (Figure 3B). Moreover, expression of *CD47*, which is considered to be a cell surface marker of primitive erythrocytes (17), was distributed in each cluster (Supplementary Figure 5). These results indicated that the cells in all the clusters were gradually transitioning through different stages of erythropoiesis. Furthermore, gene ontology (GO) analysis of biological process (BP) illustrated that cluster 0, 1, 4, and 5 had nuclear and ribosomal functions, including transcription and translation. However, the oxygen transport process was only available in cluster 2, 3, and 6. Cluster 6 additionally had heme synthesis and erythrocytes development processes (Figure 3C). Therefore, these findings revealed that the RBCs not only had nuclei but also other organelles. Based on these outcomes, we considered that the RBCs in the PBMCs from HFRS included NRBCs.

**Figure 4:**
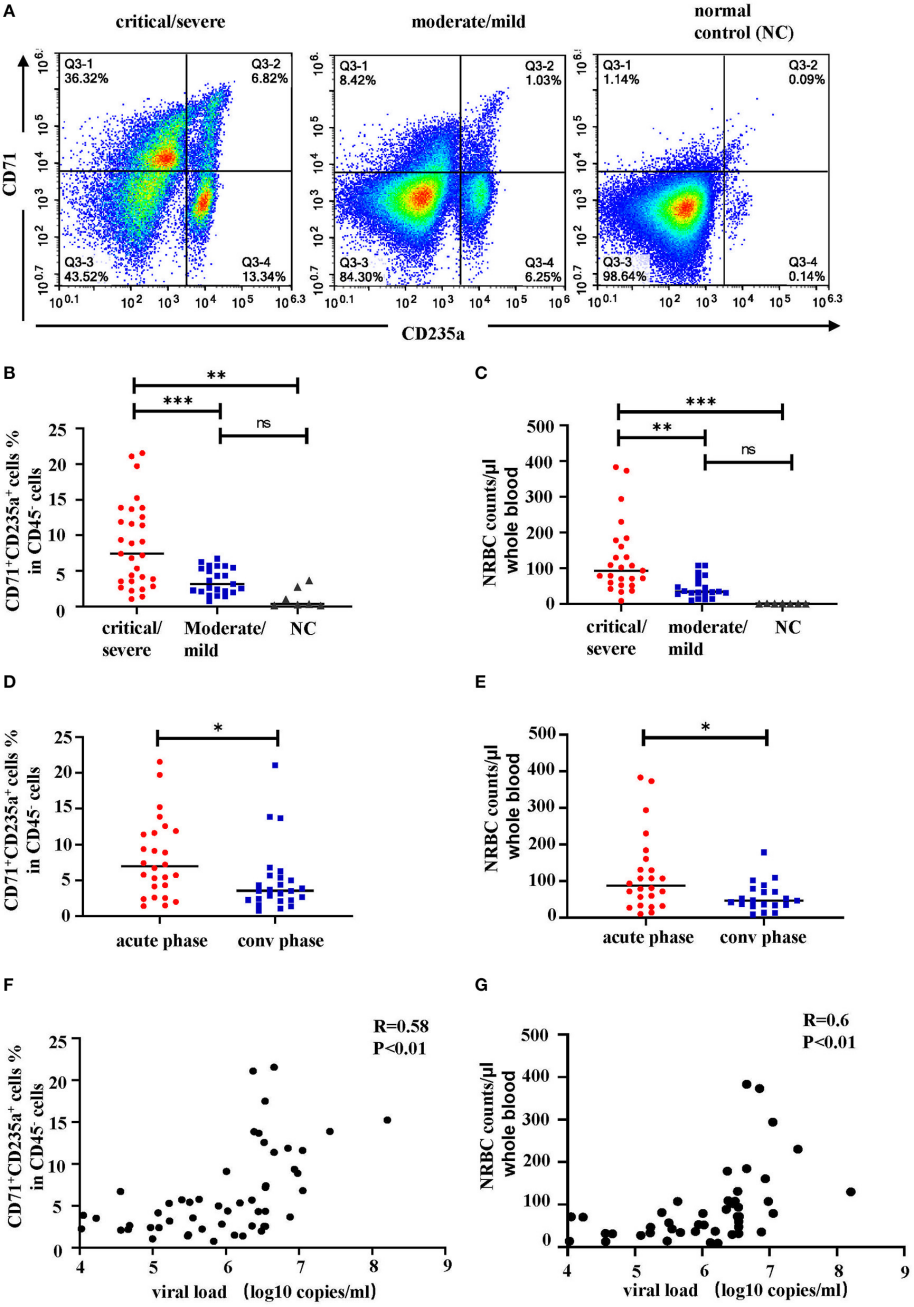
The percentage and cell counts of NRBCs detected by flow cytometry were correlated with HFRS disease severity. Representative flow cytometric graphs **(A)** and cumulative results **(B)** calculating percentage of CD71^+^CD235a^+^ cells (NRBCs) in CD45^−^ cells. Data was analyzed using One-way ANOVA. Results are presented as means ± SEM. **(C)** Comparison of NRBC total cell counts per μl whole blood in different disease severity. Data was analyzed using One-way ANOVA. Results are presented as means ± SEM. Comparison of **(D)** percentage and **(E)** cell counts of NRBCs in acute phase and convalescent (conv) phase in HFRS patients. Data was analyzed using Student *t*-test. Results are presented as means ± SEM. Analysis of correlation between viral load and percentage **(F)** or cell counts **(G)** of NRBCs by Spearman correlation. The r and *P*-values are indicated on the graph. ^*^P < 0.05, ^**^P < 0.01, ^***^P < 0.001, ns means no significant, N number equals 52 samples from 40 HFRS patients including 32 males and 8 females, N (NC) number equals 7.

To further confirm these RBCs are nucleated erythroid cells, the flow cytometry was also applied to detect CD71^+^CD235a^+^ cells, which are the erythroid lineage markers as previously reported (14), in PBMCs from 52 HFRS samples (40 HFRS patients including 29 critical HFRS samples, and 23 moderate HFRS samples) and 7 health donors (setting as normal control, NC). The results showed that the critical HFRS patients had significantly higher percentage of CD71^+^CD235a^+^ cells in their acute phase. While, the PBMCs from moderate HFRS patients and health donors did not have a discernible CD71^+^CD235a^+^ population (Figures 4A,B). The total cell counts of NRBCs were calculated by counting the PBMCs total cell numbers in 10 ml whole blood and evaluating the proportion of NRBCs in PBMCs determined by flow cytometry. As shown in Figure 4C, the samples from critical/severe patients had more NRBCs cell counts/μl whole blood than moderate/mild patients. It is also demonstrated that in acute phase of HFRS, including both critical/severe and moderate/mild patients, the proportion (Figure 4D) and total cell counts of NRBCs (Figure 4E) were higher than that in convalescent phase. In our previous study, we have demonstrated the HTNV viral load are correlated with disease severity (15). To further verify the relationship between NRBCs and HFRS severity, the correlation between the viral load and the percentage of NRBCs were calculated. As expected, the percentage (Figure 4F) and the cell counts (Figure 4G) of NRBCs were positively correlated with the HTNV viral load.

From these, we concluded that the NRBCs were present in the PBMCs of HFRS patients and were correlated with disease severity.

### The Distributions of NRBCs Clusters Were Associated With Disease Severity

**Figure 5:**
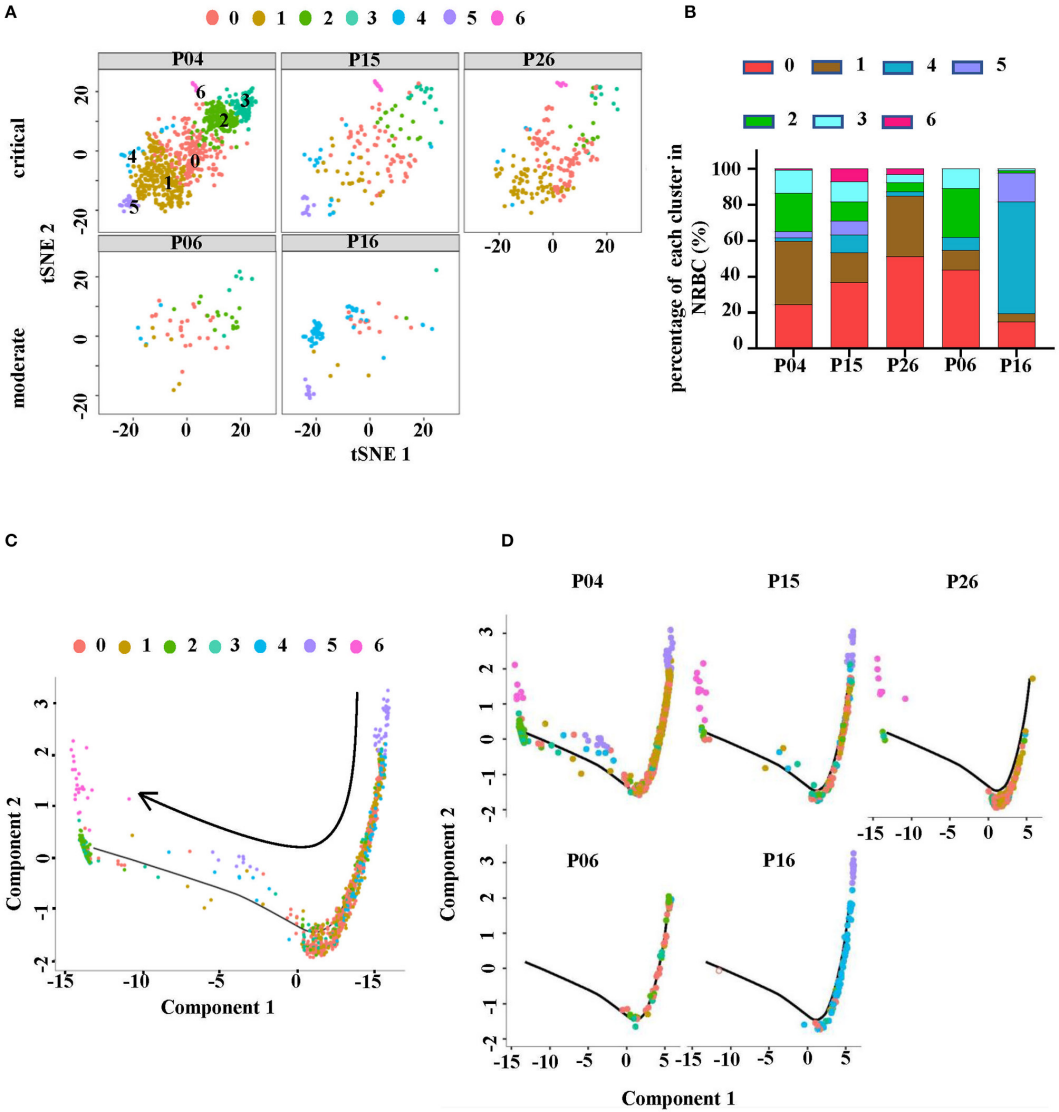
NRBC cluster distributions were different in each sample. **(A)** The tSNE plots showing the RBCs in each studied sample. The upper row represents critical HFRS patients while the lower one represents moderate HFRS patients. **(B)** A comparison of the proportion of each cluster in each sample. **(C)** The Monocle pseudotime trajectory showed the differentiation of NRBC, from the beginning on the top right and advancing as cells approached the left. **(D)** The display of pseudotime trajectory in each sample with the upper level from critical HFRS patients and lower level from moderate HFRS patients.

The distribution of these NRBCs in each studied sample was observed (Figures 5A,B). Notably, the tSNE graph showed that critical HFRS patients had more cell counts of NRBCs compared to those with moderate severity (Figure 5A). The bar graph shows the percentage of each cluster in each sample and cluster 6 was absent in the moderate HFRS patients. In addition, the percentages of cluster 0 and cluster 1 were higher in critical HFRS patients. On the contrary, clusters 4 and 5 had a higher percentage in the moderate HFRS patients (Figure 5B). Since the NRBCs are thought to be erythroblasts, the study further investigated the details of their development. Monocle2 was used to perform a pseudotime analysis to determine trajectories of differentiation by ordering cells according to their transcriptional changes. The results showed that cluster 5 was positioned at the root of the pseudotime trajectory, while cluster 6 was assigned to the end of a differentiation trajectory (Figure 5C). Additionally, pseudotime analysis in each sample showed that the NRBCs in critical HFRS patients were distributed from the early stage to the end stage during the development process. However, in moderate HFRS patients, the cells were clustered around the early stage of NRBCs development (Figure 5D). These findings therefore indicated that HTNV infection affects the development of RBCs from the early to the end stage, especially in critical HFRS patients.

### NRBCs Expressed Genes Associated With Antiviral Response

**Figure 6:**
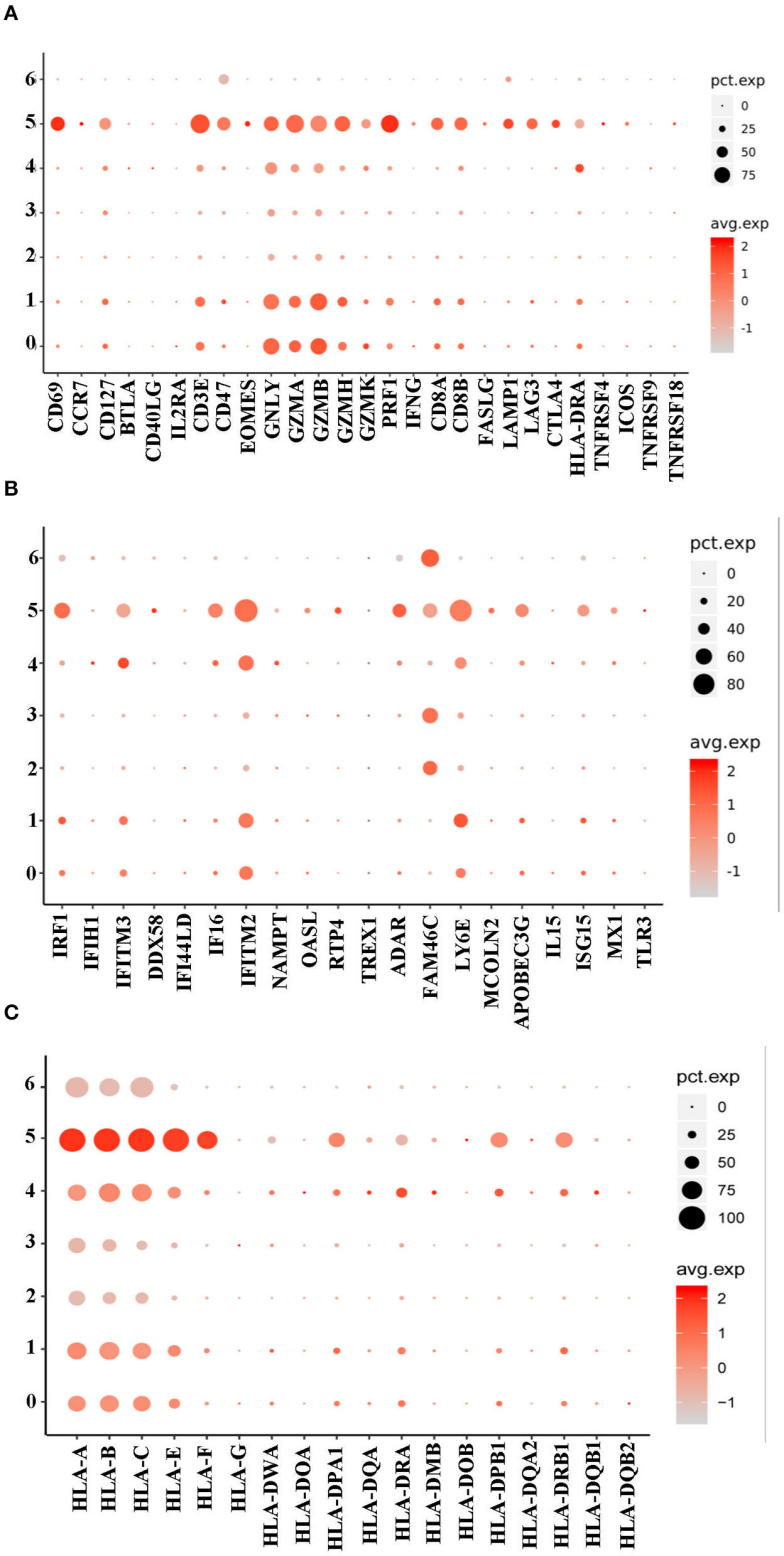
A dot plot showing that the NRBCs encoded antiviral related genes. **(A)** Cluster 0, 1, and 5 encoded higher levels of CTL related genes. **(B)** Cluster 0, 1, 4, and 5 encoded type I IFN related genes. **(C)** All the 7 clusters encoded MHC-I genes while cluster 0, 1, 4, and 5 had MHC-II genes.

The study then discussed the role of the NRBCs. Given that the normal immune related functions of mature RBCs were impaired in NRBCs, whether the cells could exert some anti-viral effects still needs to be discussed. Therefore, we profiled the expression of genes encoding antiviral markers. Notably, NRBCs from HFRS patients had higher levels of encoded genes related to CTL associated markers, such as *CD3E*, granulysin (*GNLY*), granzyme A (*GZMA*), granzyme B (*GZMB*), granzyme H (*GZMH*) and granzyme K (*GZMK*) (Figure 6A). Apart from these cytotoxic mediators, the NRBCs also encoded type-I interferon response genes including *IRF1, IFITM2, IFITM3, FAM46C* and *LY6E* (Figure 6B). Zheng *et. al* demonstrated that IFITM3 could inhibit HTNV infection (18). Moreover, the HLA class I and II molecules were widely expressed in the NRBCs (Figure 6C). Nombela also described in his review that RBCs from rainbow trout expressed MHC-I molecules on their surface. Additionally, piscine orthoreovirus infection and poly I: C were reported to stimulate the upregulation of GO pathways related to antigen processing, antigen presentation and MHC-I receptor activity (19). According to Figure 3C, GO enrichment analysis of biological processes related to viral transcription, viral life cycle and viral process were upregulated in clusters 0, 1, 4, and 5. Therefore, this data not only proved that these RBCs had nuclei but also indicated that the NRBCs might be infected by HTNV and had antiviral activity. All in all, the results suggested that NRBCs in the PBMCs from HFRS patients displayed antiviral responses.

### HTNV Infection Contributed to the Erythropoiesis Disorders in HFRS

**Figure 7:**
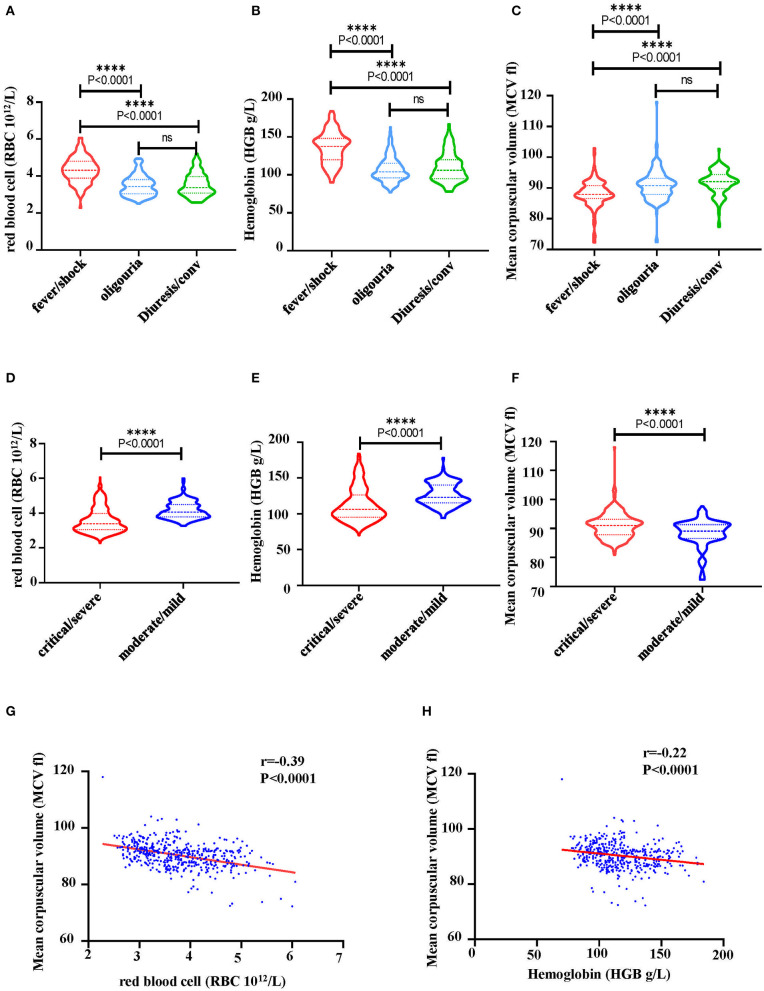
Characteristics of hematological markers in HFRS patients. **(A)** A comparison of RBC counts, **(B)** hemoglobin (HGB) and **(C)** mean corpuscular volume (MCV) at different stage of HFRS in patients. **(D)** A comparison of RBC counts, **(E)** HGB and **(F)** MCV between critical/severe and moderate/mild severe HFRS. The correlation between **(G)** RBC and MCV, **(H)** HGB and MCV was determined through Pearson correlation. The r and *P*-values are indicated on the graph. ^****^P < 0.0001; ns, no significance. N number equals 42 HFRS patients' samples including 30 males and 12 females.

Indicators of hematopoiesis, i.e., RBC, HGB and mean corpuscular volume (MCV) were then analyzed from 40 HFRS patients. It was shown that levels of RBCs (Figure 7A) and HGB (Figure 7B) gradually decreased during the HFRS process. The RBC counts in the oliguria and diuretic/convalescent stages were (3.4 ± 0.5) ×10^12^/L and (3.3 ± 0.6) ×10^12^/L, respectively. These were lower than that in the fever/shock stage (4.3 ± 0.7) ×10^12^/L and below the lower limit of the normal range (4~5.5 ×10^12^/L) (Figure 7A). Similar to the RBCs, the levels of HGB (normal range 131–150 g/L) also decreased from 137.5 ± 20.7 g/L in the fever/shock stage to 104.0 ± 16.2 g/L in the oliguria stage and 106.0 ± 19.0 g/L in the diuretic/convalescent stage, respectively (Figure 7B). Since a lot of factors might result to the presence of NRBC and a decrease in RBC and HGB, the study then explored the possible pathways that could cause the anemia. As a result, MCV and RBC were further analyzed. Although the MCV of HFRS patients did not exceeded 100 fl (femtoliter, 1×10^−15^L), it was observed that the levels increased as infection progressed. The MCV in the fever/shock stage was 87.8 ± 4.4 fl then increased to 90.8 ± 4.8 fl in the oliguria stage and 92.0 ± 4.8 fl in the diuretic/convalescent stage, respectively (Figure 7C). Moreover, the critical/severe HFRS patients had both lower RBC counts (Figure 7D) and HGB levels (Figure 7E) but higher levels of MCV (Figure 7F) compared to the moderate/mild HFRS patients. Additionally, the Pearson correlation results showed that MCV was negatively correlated with RBC and HGB, i.e., the lower the RBC counts and HGB levels, the higher the levels of MCV (Figures 7G,H). These results indicated that HFRS patients, especially those with critical/severe disease exhibited a trend of megaloblastic anemic features.

**Figure 8:**
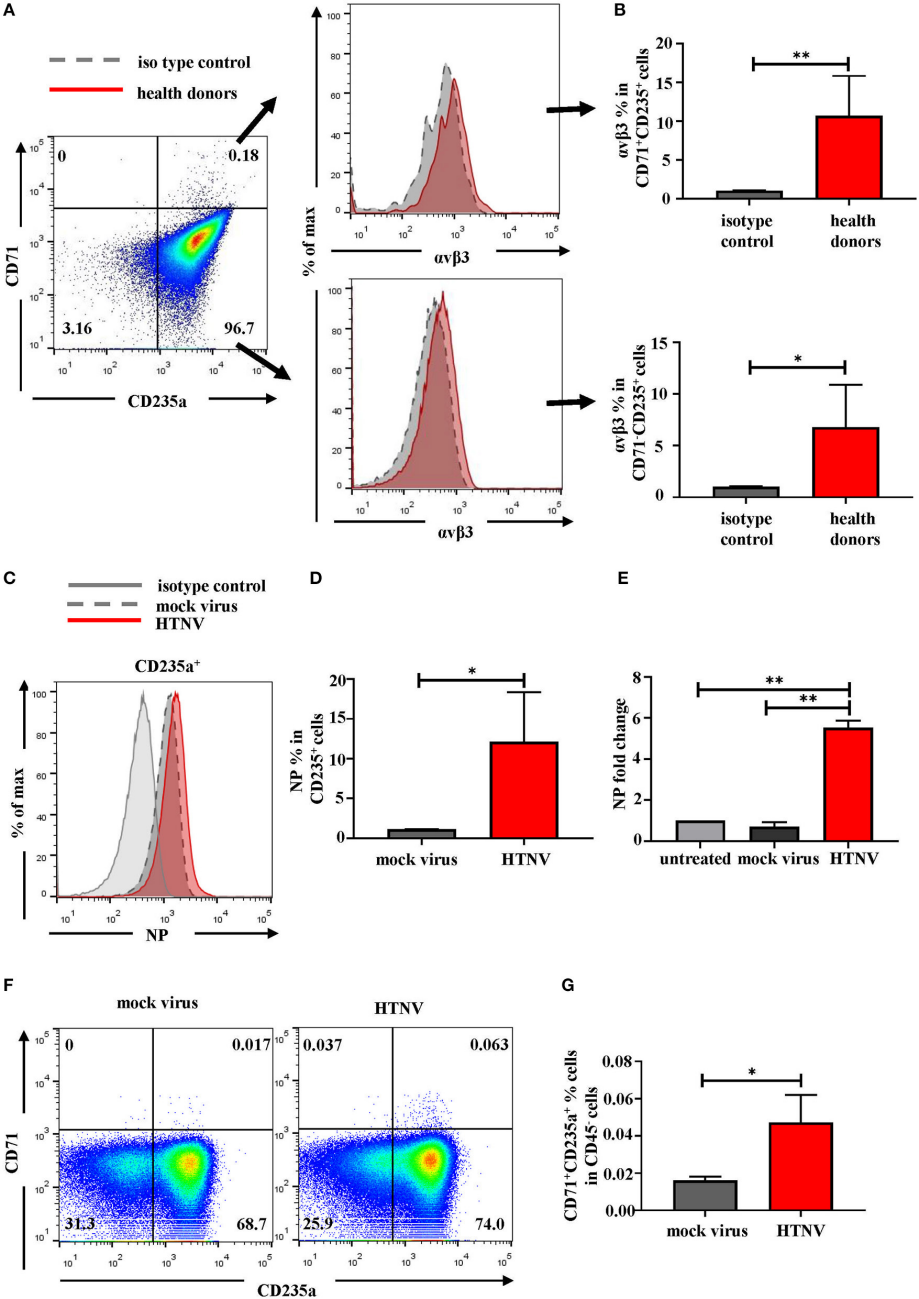
RBCs are the targets of HTNV infection. **(A)** Representative flow cytometric graphs and **(B)** cumulative results calculating percentage of αvβ3 on the surface of CD71^+^CD235a^+^ cells (upper) and CD71^−^CD235a^+^ cells (lower) from health donors. Data was analyzed using Student *t*-test. **(C)** The expression of NP in the RBCs after 72 h post infection *in vitro*. **(D)** The statistical analysis of the percentage of NP^+^ RBCs. Data was analyzed using Paired Student *t*-test. **(E)** The mRNA level of NP in the RBCs was quantified by qPCR. Data was analyzed using One-way ANOVA. Results are presented as means ± SEM. **(F)** Representative flow cytometric graphs of CD71^+^CD235a^+^ cells (NRBCs) and **(G)** comparison the percentage of NRBCs under mock infection and HTNV infection. Data was analyzed using Paired Student *t*-test. ^*^P < 0.05, ^**^P < 0.01, N number equals 4.

Based on the above analysis of clinical data, HFRS patients may have erythropoiesis disorders. According to the GO analysis of RBCs in Figure 3C, which suggested “viral life cycle and viral process” were upregulated in RBCs, we inferred that RBCs might be the target of HTNV infection. For further validation, we detected the expression of αv*β*3, which was the pathogenic receptor of HTNV (20), on the surface of RBCs. As shown in Figures 8A,B, around 10% of CD71^+^CD235a^+^ NRBCs and around 6% of CD71^−^CD235a^+^ RBCs were αv*β*3^+^. *In vitro* infection study indicated that RBCs could be infected by HTNV. The flow cytometry results suggested that 72h after infection, more than 10% of CD235a^+^ RBCs had NP in the cells (Figures 8C,D). The following qPCR results also demonstrated that the NP mRNA level was increased after HTNV infection compared to the mock virus infection (Figure 8E). It is noteworthy that *in vitro* HTNV infection could increase the percentage of CD71^+^CD235a^+^ NRBCs directly (Figures 8F,G). Based on the above results, we suggested that HTNV infection on RBCs might lead to erythropoiesis disorders in HFRS patients.

**Figure 9:**
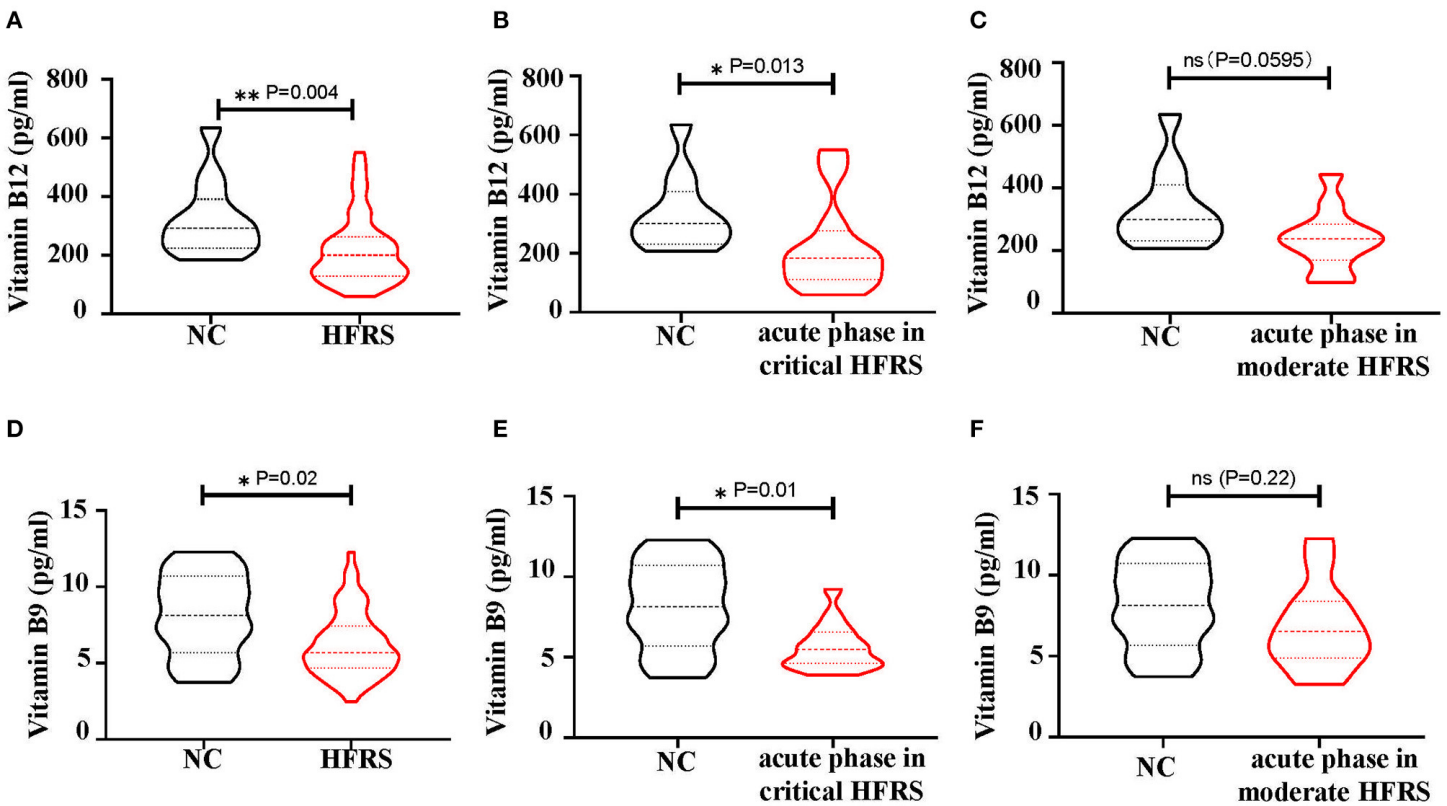
The levels of vitamin B12 and vitamin B9 in the plasma of HFRS patients. **(A)** The level of vitamin B12 and **(D)** vitamin B9 in Normal Control (NC) vs. HFRS. **(B)** The level of vitamin B12 and **(E)** vitamin B9 in NC vs. acute phase in critical HFRS. The level of **(C)** vitamin B12 and **(F)** vitamin B9 in NC vs. acute phase in moderate HFRS. The *P*-value is indicated on the graph. ^*^P < 0.05, ^**^P < 0.01; ns, no significance. N number equals 58 samples from 29 HFRS patients including 21 males and 8 females, N (NC) number equals 12.

Depending on the presence of immature NRBCs and the increase in MCV, the folate pathway, which was associated with megaloblastic anemic, was then investigated for metabolic hematopoietic abnormalities in HFRS patients. Therefore, the values of vitamin B12 (cobalamin) and B9 (folic acid), the key regulators of the folate pathway, were measured in the plasma of HFRS patients. The bar graph showed that both vitamin B12 (Figure 9A) and B9 (Figure 9D) were downregulated in HFRS patients. The study then analyzed the levels of the vitamins in each stage during HTNV infection. The findings showed that both vitamin B12 (Figure 9B) and B9 (Figure 9E) were significantly reduced in the acute phase which includes fever, shock, and oliguria in critical/severe HFRS patients. Although vitamin B12 (Figure 9C) and B9 (Figure 9F) were also decreased in the acute phase of moderate/mild HFRS patients, the differences were not significant. This data therefore suggested that HFRS patients were deficiencies in vitamin B12 and B9. The folate pathway might be a therapeutic target for treatment of the erythropoiesis disorders in HFRS patients.

## Discussion

A recent study that used scRNA-seq to profile PBMCs from COVID-19 patients suggested that RBCs were found in peripheral blood during infection (21). The study however found no significant difference in the RBCs of healthy individuals and COVID-19 patients. Notably, only one patient with acute respiratory distress syndrome (ARDS) had over 50% RBC counts, consistent with results from the present study. Additionally, patients who suffered from more severe disease had more RBCs in PBMCs. Contrary to our findings, the work on COVID-19 additionally found RBCs and neutrophils in PBMCs obtained from healthy donors. Although improper manipulation during the isolation of the PBMCs from whole blood might cause RBCs' contamination in PBMCs, the step of RBCs lysis before scRNA-seq made the results reliable. Previous studies that detected NRBCs in the whole blood of ICU patients used mechanical blood analyzers (12, 13). In these reports, patients with NRBC counts over 2,000/μl died. Therefore, the higher NRBC count, the higher mortality rate was for the ICU patients. Our study used flow cytometry to count the NRBCs, their counts could be estimated as 8–380/μl based on the percentages of NRBCs and the total PBMCs counts in whole blood.

Erythropoiesis is regulated by multiple factors including erythropoietin (EPO), iron and the folate pathway. EPO is a cytokine synthesized in the kidney to regulate erythropoiesis through binding with the EPO receptor (EPOR) expressed on the erythroid nucleated precursors (22). Therefore, deficiency of EPO is the major cause of anemia in chronic kidney disease (23). On the other hand, iron is an essential functional component of erythrocyte hemoglobin (24) and its deficiency can result in microcytic hypochromic anemia with decreased levels of RBCs, MCV < 80 fl and decreased amount of HGB (25). Moreover, vitamin B12 and vitamin B9 are the two important coenzymes for DNA synthesis in the folate pathway and deficiency of both may cause megaloblastic anemia (26). In this study, anemia [HGB < 120 g/L in premenopausal females and HGB < 130 g/L in postmenopausal females as well as males of all ages (27)] accompanied with an increasing level of MCV was observed (Figures 7G,H). However, the levels of MCV did not meet the diagnostic criteria for megaloblastic anemia. Consequently, the appearance of NRBCs in HFRS patients were related to the deficiency of vitamin B12 and/or folic acid based on the signs of megaloblastic anemia.

Furthermore, the distribution of EPOR on the NRBCs was analyzed using the NovelBrain Cloud Analysis Platform. The low expression of EPOR indicated that EPO may not have major role on the impairment of erythropoiesis during the HTNV infection process. Further investigation on the EPO level in the circulation should provide more evidence on its role in erythropoiesis during HTNV infection. Additionally, since the high expression of HBB and HBA were found at the end stage of erythrocytes and MCV did not display a declining trend, iron deficiency could not have been the main cause of impairment in erythrocyte maturation. Apart from the factors mentioned above, inflammatory cytokines such as TNF-α, IL-6 and IL-12p70 could also exert their effects on erythropoiesis (28, 29). Therefore, further studies on the role of inflammatory cytokines in erythropoiesis are needed.

Although we suggested that NRBCs in the circulating blood of HFRS patients might have antiviral effects through the expression of cytotoxic mediators, IFN response genes and MHC molecules, the role of NRBCs during infection is still unclear. Nombela et al. reported that NRBCs had immune properties including phagocytosis, antigen presentation and interleukin-like production (19). Their study also demonstrated that NRBCs could generate immune responses against viruses regardless of whether they were infected (19). However, given that CR1 was absent in the NRBCs (data not shown), it is possible that they were unable to clear the virus as normal erythrocytes, which exerted immune-clearance ability via CR1 (30). Other studies also suggested that NRBCs had immunosuppressive function during infection (14, 31). More studies are still needed to clarify the role of NRBCs in HTNV infection. In our study, we found the appearance of NRBCs during HTNV infection accompanied by the deficiency of vitamin B12 and vitamin B9 in HFRS patients. According to the previous studies that during HCV infection, vitamin B12 could inhibit HCV IRES-dependent translation and improve the rates of sustained viral response (32, 33), we suggested that the administration of vitamin supplements may confer beneficial effects to HFRS patients.

Despite the insightful findings, this study had a number of limitations. First, the sample size used for sRNA-seq was too small making it difficult to deduce statistical significance. Second, the samples were limited to the acute phase of HFRS although this is the stage where more obvious changes occur. However, changes that occurred during the convalescent phase are still unknown. Therefore, given that there is no HFRS disease model, further studies are needed to explore in detail erythropoiesis disorders in the bone marrow smears of HFRS patients.

To the best of our knowledge, this is the first study to describe changes in the RBCs of HFRS patients. It is also the first one to depict the cellular components and gene expression profiles of NRBCs in the peripheral blood of HFRS patients. Therefore, this study on RBCs may provide novel insights on adjuvant therapy and monitoring the status of patients during HTNV infection.

## Data Availability Statement

The authors declare that all data supporting the findings of this study are available within this article and its Supplementary Information files, or from the corresponding author upon reasonable request. Single-cell RNAseq gene expression data have been deposited in the Gene Expression Omnibus database (GSE161354).

## Ethics Statement

The studies involving human participants were reviewed and approved by Xijing Hospital, First Affiliated Hospital of Fourth Military Medical University, Xi'an, China, (NO. KY20173177-1). Written informed consent to participate in this study was provided by the participants' legal guardian/next of kin.

## Author Contributions

YusZ, DL, and BJ: conceived and designed the experiments. KT, HH, XJ, YM, CZ, YunZ, and RZ: recruited patients and collected and processed samples. JZ, YusZ, DL, KT, HH, YM, MW, XZ, and CZ performed the experiments. JZ, YusZ, DL, YM, and CZ: analyzed the data. DL, KT, HH, XJ, YM, YunZ, RZ, and BJ: contributed reagents/materials/analysis tools. YusZ, DL, and BJ: wrote the paper. YunZ and BJ: verified the underlying data. All authors contributed to the article and approved the submitted version.

## Conflict of Interest

The authors declare that the research was conducted in the absence of any commercial or financial relationships that could be construed as a potential conflict of interest.

## Supplementary Material

Supplementary Figure 1 Expression of genes in cluster 10 of PBMCs.

Supplementary Figure 2 Expression of genes in cluster 16 of PBMCs.

Supplementary Figure 3 A heatmap indicating the gene expression profile in cluster 0-6 of red blood cells.

Supplementary Figure 4 A heatmap indicating the gene expression module of red blood cells.

Supplementary Figure 5 Expression of CD47 in each cluster.

Supplementary Table 1 Absolute cell numbers sequenced in each sample.

Supplementary Table 2 Characteristics of enrolled subjects in testing vitamin B9/B12.
