# Supplementary figures and images for: The Presence of Circulating Nucleated Red Blood Cells Is Associated With Disease Severity in Patients of Hemorrhagic Fever With Renal Syndrome
Source: Front Med (Lausanne). 2021 May 25;8:665410. doi: 10.3389/fmed.2021.665410 (PMC8186265; doi:10.3389/fmed.2021.665410)

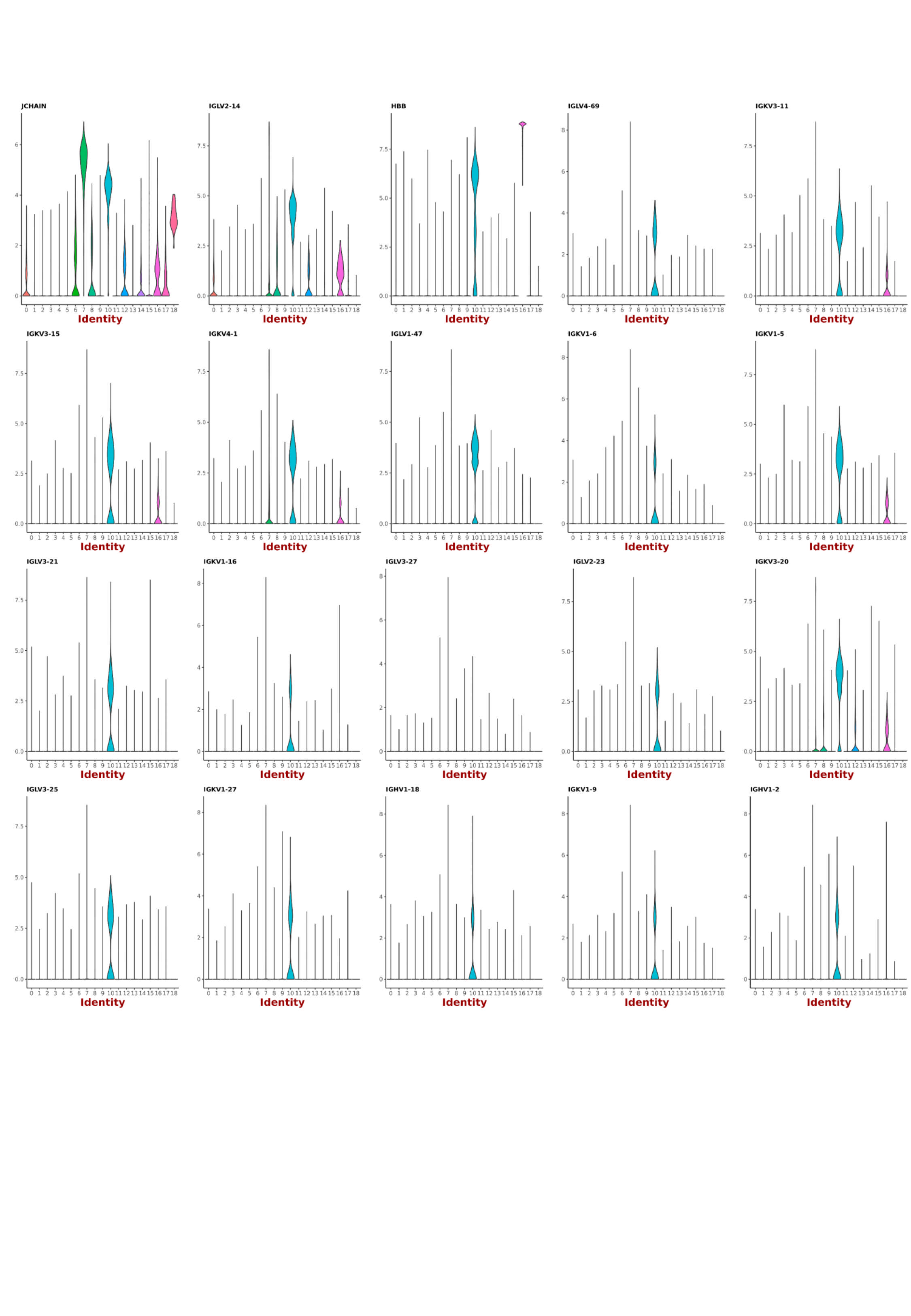

Supplement: Supplementary Figure 1 — Expression of genes in cluster 10 of PBMCs. [file Image_1.TIFF]

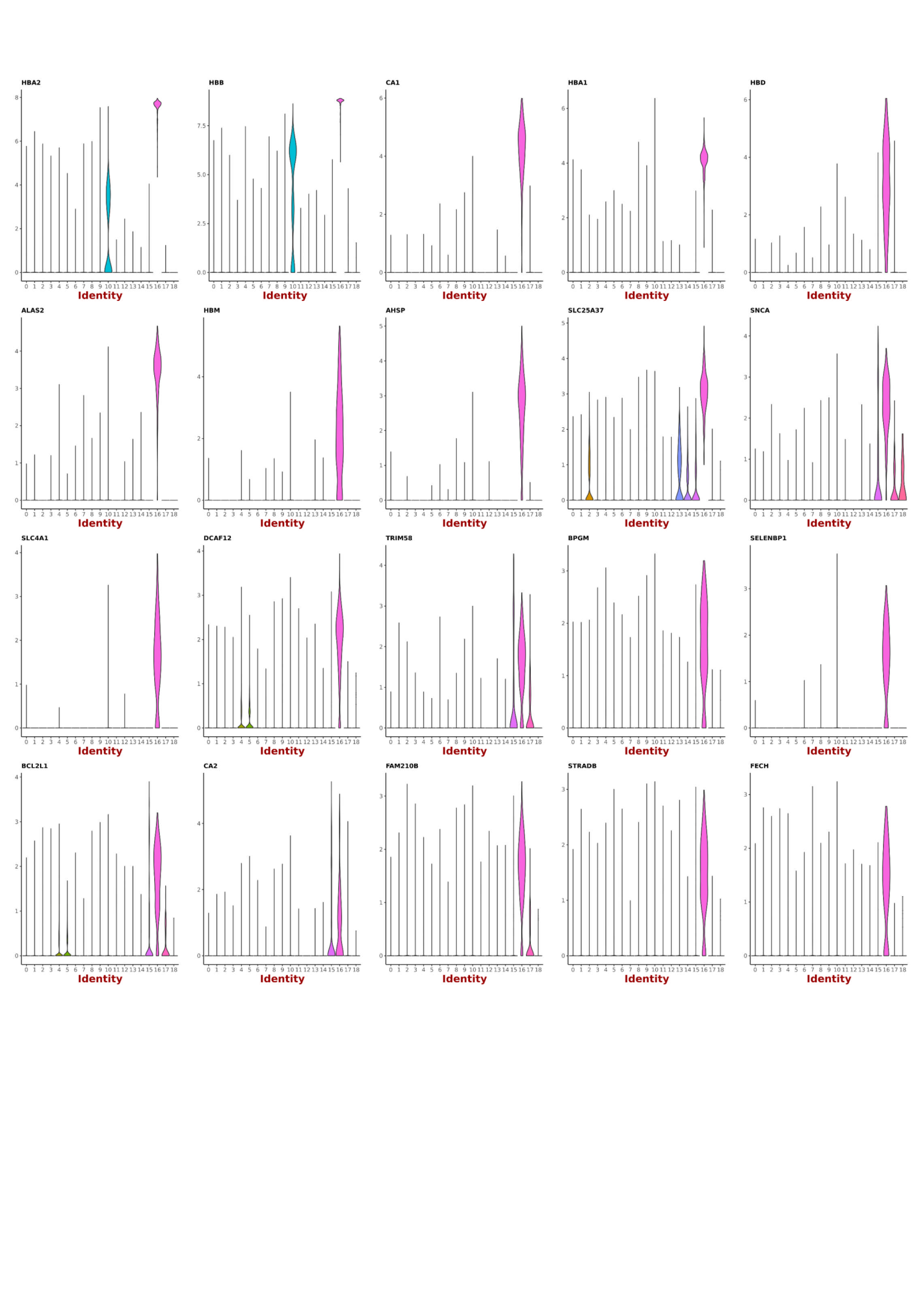

Supplement: Supplementary Figure 2 — Expression of genes in cluster 16 of PBMCs. [file Image_2.TIFF]

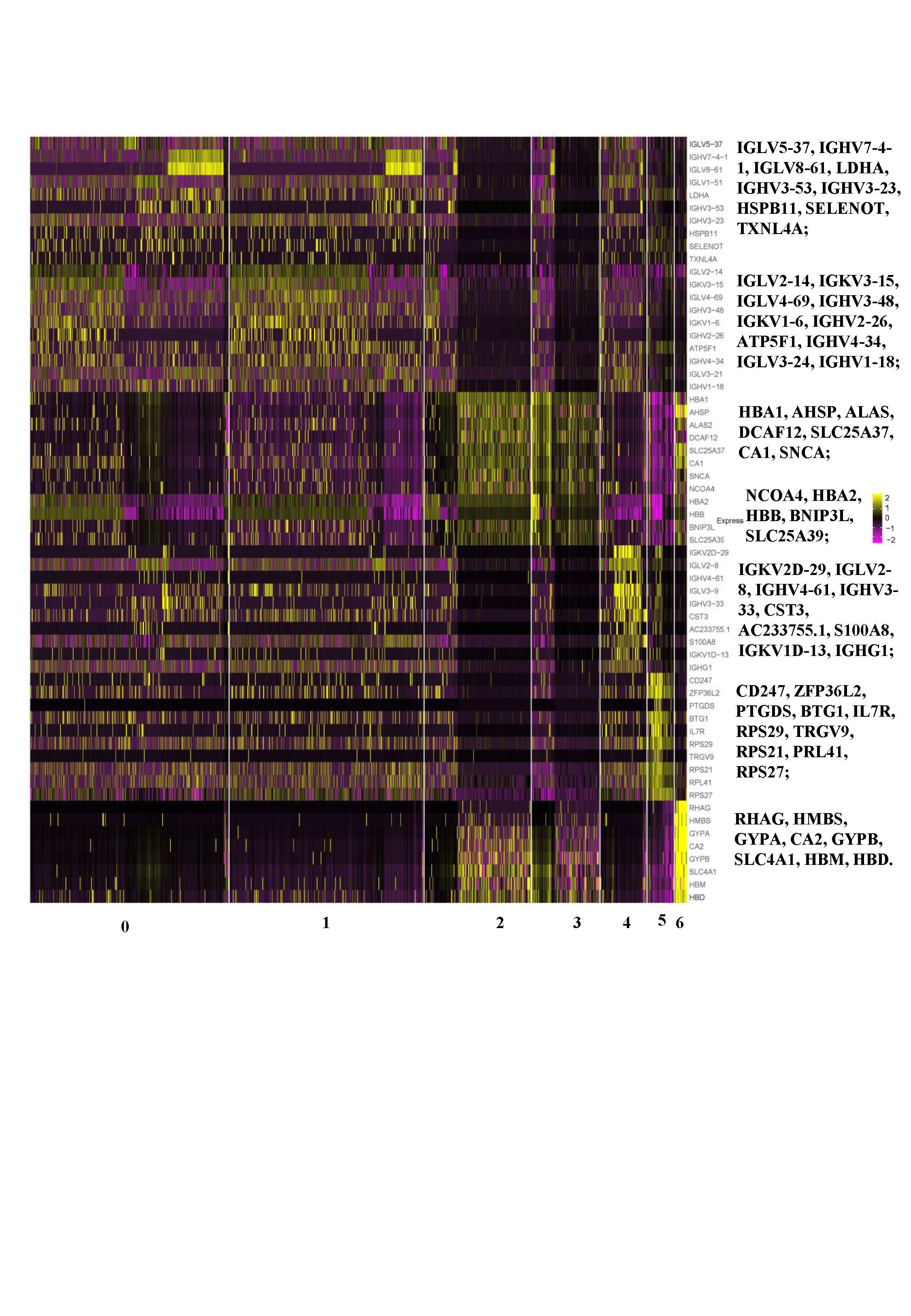

Supplement: Supplementary Figure 3 — A heatmap indicating the gene expression profile in cluster 0-6 of red blood cells. [file Image_3.TIFF]

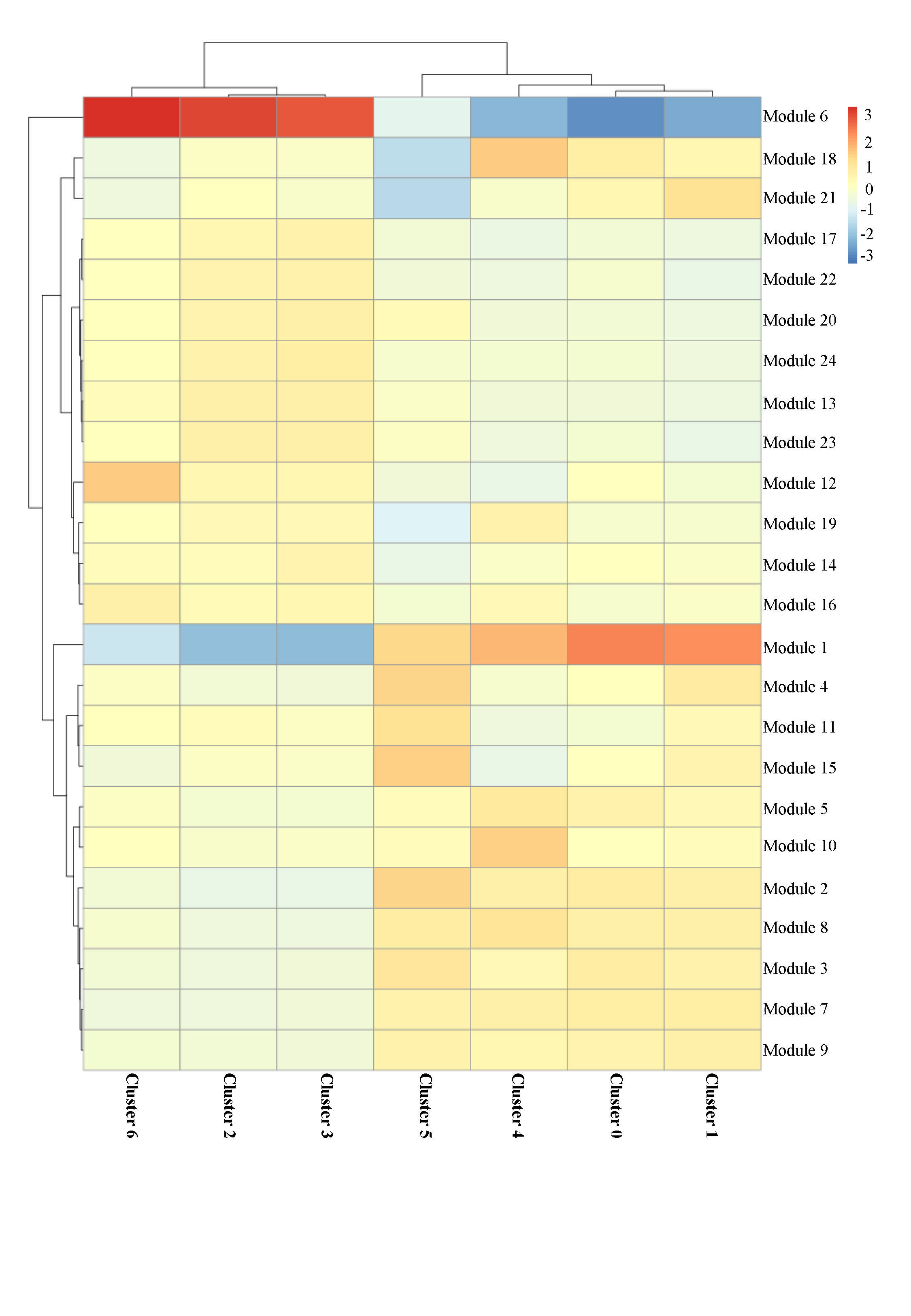

Supplement: Supplementary Figure 4 — A heatmap indicating the gene expression module of red blood cells. [file Image_4.TIFF]

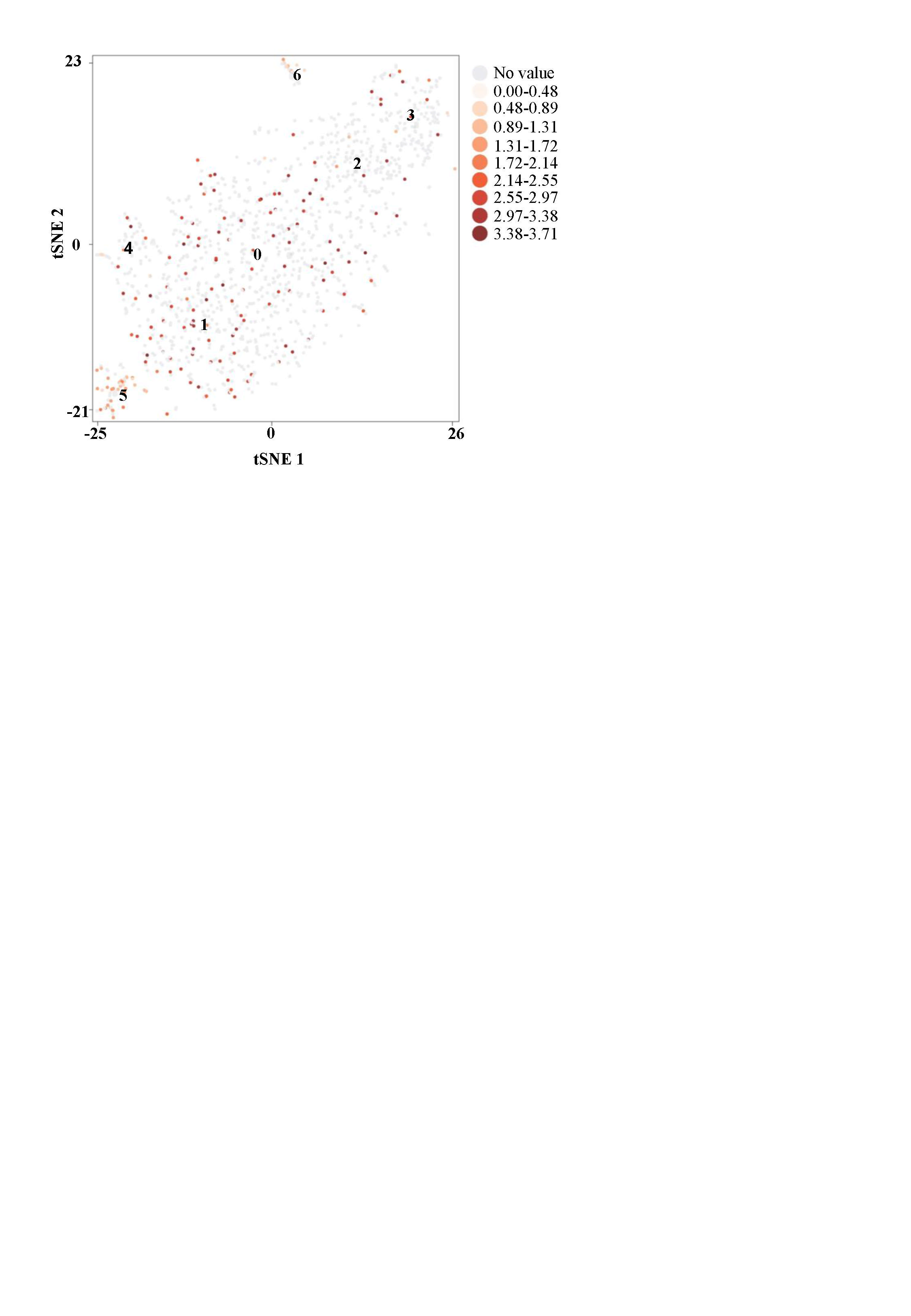

Supplement: Supplementary Figure 5 — Expression of CD47 in each cluster. [file Image_5.TIFF]
