# Supplementary material for: The Presence of Circulating Nucleated Red Blood Cells Is Associated With Disease Severity in Patients of Hemorrhagic Fever With Renal Syndrome
Source: Front Med (Lausanne). 2021 May 25;8:665410. doi: 10.3389/fmed.2021.665410 (PMC8186265; doi:10.3389/fmed.2021.665410)
Supplement: Supplementary Table 2 — Characteristics of enrolled subjects in testing vitamin B9/B12. [file Table_2.DOCX]

|  | Mild/moderate | Severe/critical | NC |
| --- | --- | --- | --- |
| **Demographic characteristics** |  |  |  |
| number | 14 | 15 | 12 |
| Age (years) | 37 (32-50) | 39 (29-55) | 36 (30-50) |
| Male (%) | 57.1% | 86.7% | 66.7% |
| **Sample number** |  |  |  |
| Acute phase (febrile/hypotensive/oliguric) | 14 | 15 | _ |
| Convalescent phase (diuretic/convalescent) | 14 | 15 | _ |

Supplementary Table 2: Characteristics of enrolled subjects in testing vitamin B9/B12.

NC: normal controls.

Values represent medians with the corresponding interquartile range.
